# Dissociated Purkinje potential as an active driver of multifocal Purkinje ventricular tachycardia: A case report

**DOI:** 10.1016/j.hrcr.2026.03.010

**Published:** 2026-03-21

**Authors:** Yusuke Hayashi, Shiho Wakasa, Shun Hirayama, Kohei Fukuda, Tomoya Yanagishita, Daiju Fukuda

**Affiliations:** Department of Cardiovascular Medicine, Osaka Metropolitan University Graduate School of Medicine, Abenoku, Osaka Japan

**Keywords:** Purkinje, Ventricular tachycardia, Dissociated Purkinje potential, Catheter ablation, Source–sink mismatch

**Key Teaching Points**

• In focal Purkinje ventricular tachycardia (VT), the earliest Purkinje potential preceding QRS onset during VT is generally the appropriate ablation target.
• Although Purkinje potentials recorded during VT are typically expected to precede the QRS complex during sinus rhythm, this criterion does not always apply.
• Purkinje potentials that appear electrically dissociated from surrounding myocardial activity during sinus rhythm may still contribute to VT initiation and maintenance and should not automatically be considered bystanders.
• Selective pacing of a Purkinje potential to determine whether it reproduces the clinical VT QRS morphology is a useful strategy to distinguish an ablation target from bystander activity.

## Introduction

Most ventricular tachycardias (VTs) in structural heart disease are scar-related. In contrast, Purkinje-related VT accounts for approximately 5%–9% of VT ablation procedures in patients with structural heart disease and is characterized by distinct electrophysiological properties and ablation strategies compared with scar-related VT.^1^^,^^2^ In focal Purkinje VT, the earliest Purkinje potential that precedes QRS onset during VT is considered the ablation target, and this potential should also precede the QRS complex during sinus rhythm, thereby acting as an important diagnostic feature.^1^ Previous experimental and clinical studies have demonstrated that electrical dissociation of the Purkinje system from the ventricular myocardium can terminate polymorphic VT.^3^ Purkinje network plays a critical role in the initiation and maintenance of VT and ventricular fibrillation.^4^^,^^5^ However, to date, there have been no reports demonstrating that a Purkinje potential appearing completely dissociated from surrounding myocardial activity during sinus rhythm directly contributes to VT initiation and maintenance. Here, we report a case of focal Purkinje VT in which a Purkinje potential recorded during sinus rhythm, apparently electrically dissociated from the surrounding myocardium, was involved in the initiation and maintenance of VT.

## Case report

**Figure 1:**
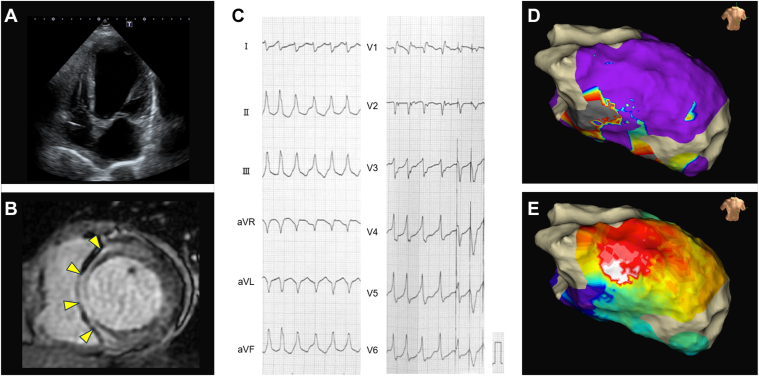
Multimodality imaging and electrophysiological findings in a patient with ventricular tachycardia storm. **A:** Transthoracic echocardiography showing severely reduced left ventricular systolic function. **B:** Cardiac magnetic resonance imaging showing extensive mid-myocardial late gadolinium enhancement in the interventricular septum (*yellow arrows*). **C:** Electrocardiographic recordings during a ventricular tachycardia (VT) storm. **D:** Left ventricular voltage map obtained during atrial pacing, showing a scar area at the basal interventricular septum corresponding to the ablation sites from the prior procedure (voltage cutoff: 0.5–1.5 mV). **E:** Activation map obtained during atrial pacing, demonstrating conduction block of the left posterior fascicle.

A 79-year-old man with dilated cardiomyopathy and a history of sustained VT had previously undergone implantation of an implantable cardioverter-defibrillator (ICD) 5 years earlier; a transthoracic echocardiographic analysis revealed diffuse left ventricular hypokinesis with a left ventricular ejection fraction of 23% (Figure 1A). Cardiac magnetic resonance imaging revealed extensive mid-myocardial late gadolinium enhancement in the interventricular septum (Figure 1B). Because of frequent ICD therapies, VT ablation was performed at another institution. During the electrophysiological study, 5 distinct VTs were documented, all of which caused hemodynamic collapse, precluding detailed mapping. Ablation was performed for a focal VT originating from the left ventricular septum, which was the only VT amenable to mapping, targeting the site of earliest ventricular activation. However, VT recurred 2 days later, progressing to a VT storm. Despite antiarrhythmic drug therapy and deep sedation, VT remained uncontrolled, resulting in hemodynamic deterioration that required mechanical ventilation and venoarterial extracorporeal membrane oxygenation (VA ECMO). The patient was transferred to our institution for repeat ablation 3 days after the initial ablation procedure. On admission, the patient’s blood pressure was 90/52 mmHg; his heart rate 70 beats/min; and his oxygen saturation 98%. Chest radiography showed no evidence of pulmonary congestion. The resting electrocardiogram demonstrated atrial pacing with ventricular sensing, and the intrinsic QRS morphology exhibited a right bundle branch block pattern. An incessant VT with a qR pattern in lead V1 and an inferior axis was observed, prompting emergent catheter ablation (Figure 1C).

**Figure 2:**
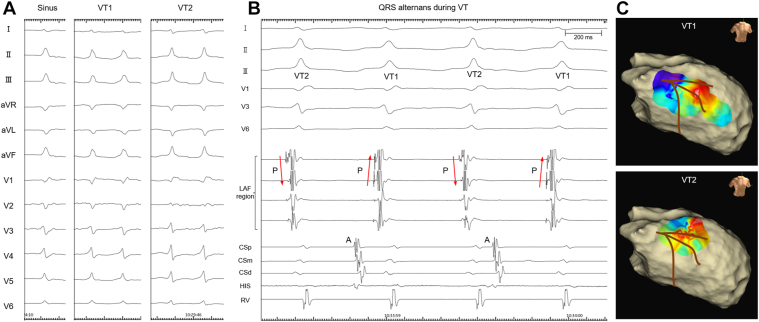
Electrocardiographic and activation mapping characteristics of VT1 and VT2. **A:** QRS morphologies during sinus rhythm and during VT1 and VT2. The QRS morphology of VT1 closely resembles that observed during sinus rhythm. **B:** Both VT1 and VT2 exhibit QRS alternans. Although the earliest Purkinje potentials are recorded in the left anterior fascicular region in both VTs, their activation sequences differ (*red arrows*). **C:** Activation maps annotate with Purkinje potentials during VT1 and VT2. The *brown line* indicates the sites where fascicular potentials were recorded during sinus rhythm. CS = coronary sinus; LAF = left anterior fascicle; RV = right ventricle; VT = ventricular tachycardia.

**Figure 3:**
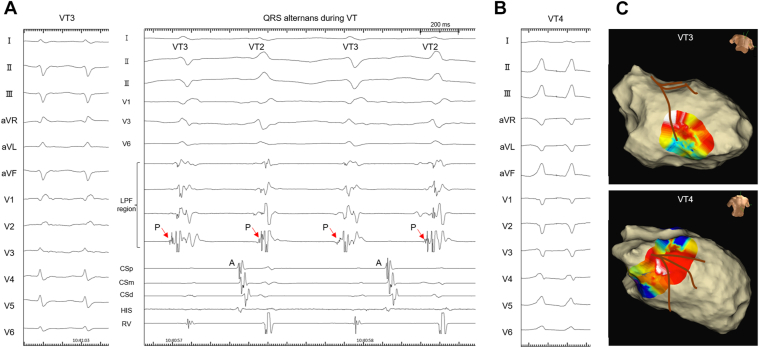
Electrocardiographic and activation mapping characteristics of VT3 and VT4. **A:** VT3 demonstrating a right bundle branch block morphology with a superior axis. QRS alternans is also observed between VT2 and VT3. During VT3, the earliest Purkinje potential is recorded in the left posterior fascicular region and preceded QRS onset by 40 ms. **B:** VT4 demonstrating a left bundle branch block morphology with an inferior axis. **C:** Activation maps annotate with Purkinje potentials during VT3 and VT4. The *brown line* indicates the sites where fascicular potentials were recorded during sinus rhythm. CS = coronary sinus; LPF = left posterior fascicle; RV = right ventricle; VT = ventricular tachycardia.

An electrophysiological study was performed using the EnSite X mapping system under VA ECMO support because of hemodynamic instability. A left ventricular voltage map created during atrial pacing demonstrated a scar area at the basal interventricular septum (voltage cutoff: 0.5–1.5 mV) (Figure 1D), corresponding to the ablation site from the previous session. The clinical VT (VT1) was not inducible by programmed stimulation but occurred incessantly, suggesting a non-reentrant mechanism. During VT1, a Purkinje potential preceding ventricular activation was recorded in the left anterior fascicle region, and the preceding Purkinje–Purkinje (PP) interval determined the subsequent ventricular–ventricular (VV) interval, consistent with Purkinje VT. QRS alternans was observed during VT, with marked beat-to-beat morphological changes in lead V1 (VT2) (Figure 2). Although Purkinje potentials preceded ventricular activation in all VTs, their activation sequences differed. Another VT (VT3) occurred spontaneously and also exhibited QRS alternans. During VT3, a Purkinje potential preceding QRS onset by 40 ms was recorded in the left posterior fascicular region, and the PP interval again dictated the VV interval, leading to a diagnosis of left posterior fascicular Purkinje VT (Figure 3A).

**Figure 4:**
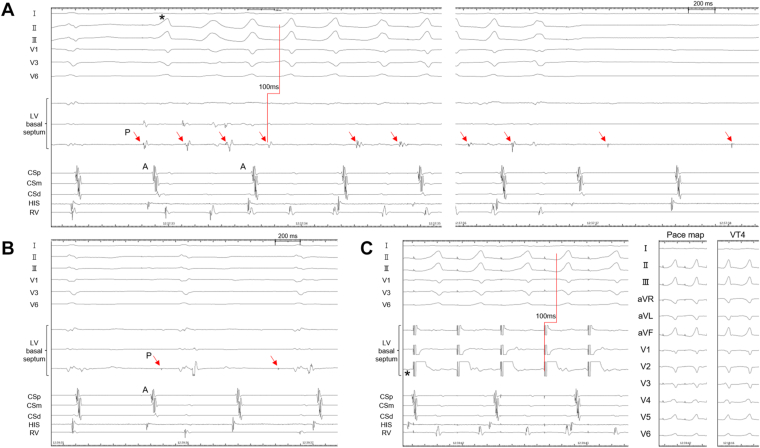
Dissociated Purkinje potential as an active driver of VT4 demonstrated by selective capture pacing. A: VT4 is initiated by a PVC, A: VT4 is initiated by a PVC. A Purkinje potential preceding the trigger PVC (asterisk) is recorded (red arrow); this potential precedes QRS onset by 100 ms during VT. After termination of VT, this potential persists as a regular dissociated activity. B: This Purkinje potential is completely dissociated from both atrial and ventricular activation. C: Pacing at the site where the dissociated Purkinje potential is recorded (asterisk), at the same cycle length as VT4, successfully achieves selective capture of the Purkinje potential. QRS complexes are generated with a latency of 100 ms and are identical in morphology to those observed during VT4. CS = coronary sinus; LV = left ventricle; PVC = premature ventricular complex; RV = right ventricle; VT = ventricular tachycardia.

Activation maps annotated to Purkinje potentials demonstrated completely different earliest Purkinje activation sites and sequences among VT1, VT2, and VT3, consistent with multifocal Purkinje VT. Radiofrequency ablation at each earliest Purkinje activation site rendered VT1–3 non-inducible. Subsequently, a new VT (VT4) occurred spontaneously (Figure 3B). During VT4, a high-frequency potential preceding QRS onset by approximately 100 ms was recorded at the basal left ventricular septum. Transient atrioventricular block occurred when VT4 terminated spontaneously (Figure 4A). Moreover, the same high-frequency potential continued to be recorded after VT termination and was completely dissociated from both atrial and ventricular activities (Figure 4B). As a Purkinje potential was recorded at this site during sinus rhythm, this potential was considered a dissociated Purkinje potential. Notably, selective pacing capture of this potential at the same cycle length as VT4 using a pacing output of 5 V at a pulse width of 1 ms reproduced an identical QRS morphology to VT4 with a distinct latency of 100 ms (Figure 4C). A single radiofrequency application at this site eliminated VT4, and no further VT was inducible. For each morphological characteristic of VT, radiofrequency ablation was targeted at the site that showed the earliest Purkinje potential preceding the VT QRS complex, thus indicating that the Purkinje network itself served as the arrhythmogenic substrate rather than the surrounding ventricular myocardium. Complete atrioventricular block developed after the procedure; however, the patient was successfully weaned from mechanical ventilation and VA ECMO and was upgraded to a cardiac resynchronization therapy defibrillator. No VT recurrence was observed during 10 months of follow-up.

## Discussion

This case represents multifocal Purkinje VT with QRS alternans, in which a dissociated Purkinje potential, seemingly a bystander, was critical in the initiation and maintenance of VT4. This finding is clinically important as it demonstrates that a Purkinje potential seemingly dissociated from surrounding myocardial activity can still serve as an arrhythmogenic substrate. Although a non-reentrant mechanism was suspected based on the incessant nature of VT and electrophysiological findings, it could not be definitively proven because selective entrainment pacing of the Purkinje potential during VT could not be achieved.

In focal Purkinje VT, the earliest Purkinje potential preceding ventricular activation during VT is considered the ablation target and is generally expected to precede the QRS complex during sinus rhythm.^1^ Although abnormal Purkinje fibers located within low-voltage areas or scar border zones are known to contribute to post–myocardial infarction ventricular arrhythmias,^5^ involvement of a Purkinje potential completely dissociated from surrounding myocardium has not been previously reported. In the present case, dissociated Purkinje activity was recorded at a slow rate after termination of VT4 and showed no conduction to the ventricular myocardium during sinus rhythm. However, selective capture of the Purkinje potential at the VT4 cycle length resulted in conduction to the ventricular myocardium and reproduced the identical VT QRS morphology. This phenomenon can be explained by source–sink mismatch. At the Purkinje–muscle junction, a conduction delay of approximately 10 ms is typically observed; this delay reflects the time required for current supplied by the Purkinje fibers to depolarize the ventricular myocardium, representing a large electrical load (sink). When conduction at the Purkinje–muscle junction becomes excessively slow, prior experimental studies have demonstrated that source–sink mismatch progressively worsens over time, ultimately rendering the current-supplying capacity of the Purkinje fibers relatively insufficient.^6^, ^7^, ^8^ In the present case, the low activation rate during sinus rhythm may have limited the source current from the Purkinje fibers, preventing effective depolarization of the ventricular myocardium. Conversely, high-rate pacing likely increased the source current, enabling successful myocardial capture.

This case underscores that dissociated Purkinje potentials should not automatically be regarded as bystanders. Even when Purkinje activity appears electrically dissociated from surrounding myocardial activation, it may be indicative of an active arrhythmogenic substrate in focal Purkinje VT, thereby making careful electrophysiological assessment essential. Specifically, selective pacing of the Purkinje potential and confirmation that the paced QRS morphology precisely reproduces the clinical VT provide critical evidence for identifying the true ablation target and avoiding unnecessary or excessive myocardial ablation.

## Conclusion

Dissociated Purkinje potentials can be an active ablation target rather than bystander activity in focal Purkinje VT. Selective capture of the Purkinje potential with reproduction of the clinical VT QRS morphology is important for guiding appropriate and effective ablation.

## Disclosures

The authors have no conflicts of interest to disclose.
